# Comprehensive survey of p94/calpain 3 substrates by comparative proteomics – Possible regulation of protein synthesis by p94

**DOI:** 10.1002/biot.200700018

**Published:** 2007-05

**Authors:** Yasuko Ono, Chikako Hayashi, Naoko Doi, Fujiko Kitamura, Mayumi Shindo, Kenichi Kudo, Takuichi Tsubata, Mitsuaki Yanagida, Hiroyuki Sorimachi

**Affiliations:** Department of Enzymatic Regulation for Cell Functions (Calpain Project), The Tokyo Metropolitan Institute of Medical Science (Rinshoken)Tokyo, Japan; Department of Applied Biological Science, Faculty of Science and Technology, Tokyo University of ScienceChiba, Japan; CREST, Japan Science and Technology (JST)Saitama, Japan; Proteomics & Small Molecules Division, Applied Biosystems Japan Ltd.Tokyo, Japan; KYA TECH CorporationTokyo, Japan; Institute for Environmental and Gender Specific Medicine, Juntendo University Graduate School of MedicineChiba, Japan

**Keywords:** Calpain, Fodrin, iTRAQ, Substrate, Proteolysis

## Abstract

Calpain represents a family of Ca^2+^-dependent cytosolic cysteine proteases found in almost all eukaryotes and some bacteria, and is involved in a variety of biological phenomena, including brain function. Several substrates of calpain are aggressively proteolyzed under pathological conditions, e.g., in neurodegenerating processes, fodrin is proteolyzed by calpain. Because very small amounts of substrate are proteolyzed by calpain under normal biological conditions, the molecular identities of calpain substrates are largely unknown. In this study, an extensive survey of the substrates of p94/calpain 3 in COS7 cells was executed using iTRAQ™ labeling and 2-D LC-MALDI analysis. p94 was used because: (i) several p94 splicing variants are expressed in brain tissue even though p94 itself is a skeletal-muscle-specific calpain, and (ii) it exhibits Ca^2+^-independent activity in COS cells, which makes it useful for evaluating the effects of p94 protease activity on proteins without perturbing the cells. Our approach revealed several novel protein substrates for p94, including the substrates of conventional calpains, components of the protein synthesis system, and enzymes of the glycolytic pathway. The results demonstrate the usefulness and sensitivity of this approach for mining calpain substrates. A combination of this method with other analytical methods would contribute to elucidation of the biological relevance of the calpain family.

## 1 Introduction

Calpain (Clan CA, family C02, EC 3.4.22.17) comprises a family of cysteine proteases whose activity is dependent on an increase in cytosolic Ca^2+^ [1–3]. In the human genome, 14 genes encode calpain homologues. They are categorized into two groups, ubiquitous and tissue-specific calpains, based on their expression profiles. Because the two ubiquitous calpains, μ- and m-calpain, were the first calpains to be identified, the term ‘conventional calpains’, was applied to them. They are heterodimers and consist of large catalytic subunits (CAPN1 and CAPN2, or μCL and mCL) and smaller regulatory subunits (30K sub-units). As protease enzymes, the μ- and m-calpains have common substrate and inhibitor specificities but differ in their Ca^2+^ dependence and preference for peptide substrates over protein substrates. Unexpectedly, it was shown that a lack of m-calpain, but not μ-calpain, causes embryonic lethality [4–7]. Therefore, despite an activation requirement of Ca^2+^ concentrations of several hundred millimolar, the implementability of which is yet to be demonstrated *in vivo*, it has been proposed that m-calpain plays a fundamental role in the maintenance of life and/or is critical for early development, and is capable of compensating for some μ-calpain functions.

Under normal cellular conditions, calpain activation is a tightly regulated process coupled to the homeostasis of the intracelluar free calcium concentration ([Ca^2+^]_i_). Calpain is activated when [Ca^2+^]_i_ concentrations increase. The cleavage of target proteins by calpain often alters their functions, activities, and/or structures, modulating many cellular functions. Therefore, identification of calpain substrates and elucidation of the effects of calpain proteolysis on their functions is important for elucidation of the molecular mechanisms of biological phenomena that involve calpain. As calpain is important for normal cell function, many pathological conditions are associated with over-activation of calpain consequent to loss of [Ca^2+^]_i_ homeostasis. For example, in brain ischemia, neu-ronal cell death, cardiac dysfunction, and muscular dystrophies, excessive proteolysis of substrates by calpain, which is not the primary cause of these pathological states, further compromises cellular functions and aggravates symptoms [8–13]. These conditions indicate that calpain is a promising target for strategies aimed at ameliorating and/or retarding the progression of the symptoms of these diseases [14–16].

On the other hand, only one human pathological condition has been shown to result from genetic loss of calpain protease activity. Limb-girdle muscular dystrophy type 2A (LGMD2A) is caused by a loss of the protease activity of the skeletal-muscle-specific calpain p94/calpain 3, which is caused by a pathogenic mutation of the p94 gene, *CAPN3* [17, 18]. Studies on the properties of the p94 molecule and on specific cellular events involving p94, such as apoptosis and myogenic differentiation, have contributed to current understanding of the p94 molecule, and several proteins have been identified as substrates of p94 [19–21]. Although the role that the p94 molecule plays *in vivo* is not fully understood, it is possible that the dys-trophic muscle phenotype observed in LGMD2A is the result of accumulated losses in cellular responses to various physiological perturbations because p94 lacks its protease activity. An alternative ubiquitous promoter of *CAPN3* was recently identified and the expression of several p94 variants in various organs, including the brain, has been reported [22–24], suggesting that p94 is important for tissues other than skeletal muscles.

The primary structure of p94 is very similar to that of the catalytic subunits of the μ- and m-calpains; however, several unique and enigmatic properties distinguish p94 from the conventional calpains. Most significantly, p94 exhibits very rapid autoproteolytic activity in all protein expression systems examined to date [25, 26]. Although native p94 protein is abundant in protein preparations made from skeletal muscles, it disappears as a result of autolysis during purification, suggesting that a skeletal-muscle-specific mechanism for stabilizing p94 prevails *in vivo* [27]. As a consequence of its autolysis, analysis of p94 protease activity using standard methodologies is difficult. To elucidate the etiology of LGMD2A, data are required on the identity of the substrates of p94 and on when and how they are proteolyzed. As our previous study [28] demonstrated that p94 has substrate specificities very similar to those of the μ- and m-calpains, studies on p94 substrates would contribute to the understanding of brain-specific diseases that involve the conventional calpains.

One of the advantages of using p94 is that Ca^2+^ stimulation is not necessary for its activation; the conventional calpains have to be activated by an increase of [Ca^2+^]_i_ concentrations provoked by molecules such as Ca^2+^ ionophores, the presence of which affects the entire cell. Using COS7 cells transfected with a p94 expression vector and Western blot analyses, we previously detected proteolysis of several proteins, suggesting that p94 prote-olytic activity is readily exerted and that the proteins identified are potential *in vivo* p94 substrates [17]. Considering that more than 100 proteins have been identified as *in vitro* substrates of conventional calpains [1], many p94 substrates have not yet been identified because of the constraints of conventional detection methods.

In this study, quantitative proteomic analysis was performed using the COS7 expression system and the iTRAQT™ method [29–31]. The isobaric tagging of pep-tides using multiplexed iTRAQT™ reagents prior to MS allows identification and quantification of the same peptide derived from different origins, *e.g.*, cancer cells at different malignant states. Proteases represent one of the most direct effectors on the amount of substrate proteins. Therefore, quantitative as well as comprehensive proteomic analysis is promising for identification of novel protease substrates, although few reports have been published [32]. Several proteins were identified as potential p94 substrates; these included known substrates of conventional calpains, which supports that p94 and the conventional calpains have common substrate specificities, an indication from our previous study [28]. By Western blot analysis, fodrin, one of the few *in vivo* substrates of conventional calpains that is proteolyzed during ischemia, was readily detected to be proteolyzed by p94. The net abundance of fodrin determined by its trypsin-di-gested product did not decrease in concert with the decrease in unproteolyzed fodrin levels, suggesting that the proteolytic product by p94 is relatively stable. These data highlight the usefulness of proteomic analysis as a complement to conventional analytical methods for thorough analysis of proteolytic events elicited by calpain.

## 2 Materials and methods

### 2.1 Protein expression in COS7 cells and iTRAQT™ reagent labeling

Cell culture and transfection (electroporation) methods for COS7 cells, cDNA constructs for expressing human p94s, wild-type (WT) and a protease-inactive p94:C129S mutant (CS) were described previously [27]. At 72 h after electroporation, harvested cells (*ca.* 0.5 × 10^7^−1.0 × 10^7^ cells) were lysed in 200 μL 20 mM triethylammonium bicarbonate (Nacalai Tesque, Japan) by sonication. The cell lysate was centrifuged at 18 000 × *g* for 20 min at 4°C, the supernatant was removed, and protein concentration was quantified using the DC protein assay (Bio-Rad, Japan).

**Figure 1:**
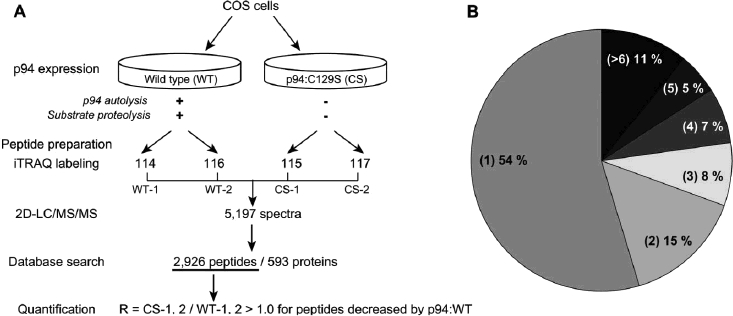
Proteomic analysis of COS7 cells expressing p94. (A) Overview of experimental design. (B) Number of peptides/proteins identified in the 2-D LC-MALDI analysis. More than half the identified proteins were represented by a single peptide. Of the proteins identified, 11% had more than six peptides; these included vimentin, several different heat shock proteins, and cytoplasmic actins, which are known to be abundant.

From cells expressing either WT or CS, 72 μg protein was prepared and diluted to 20 μL using 0.5 M triethylammonium bicarbonate containing 0.1% SDS. Protein was reduced, alkylated, and trypsin-digested prior to labeling with iTRAQT™ reagent according to the manufacturer's protocol (ABI, USA). The digested protein prepared from each cell was divided into two portions for labeling with iTRAQT™ 114 and 116 (WT) or 115 and 117 (CS) (Fig. 1A).

### 2.2 Peptide fractionation for 2-D LC-MALDI-MS/MS analysis

2-D peptide fractionation was performed on a DiNa Direct Nano-flow LC system equipped with a MALDI-plate spotter (DiNa MaP; KYA Tech, Japan ) using a strong cation exchange (SCX) column [HiQ Sil SCX, 0.5 mm inside diameter (id) × 35 mm], a reverse-phase (RP) trap column (HiQ Sil C18-3, 0.8 mm id × 3 mm), and an RP analytical column (HiQ Sil C1 8-3, 0.15mm id×50 mm). Labeled peptides were pooled, diluted with 2.5 volumes of MilliQ water, and acidified to pH 3.0 using 1 M phosphoric acid. Half of the preparation was used per injection. Peptides trapped on the SCX column were eluted using a gradient program consisting of eight linear segments: 0–5% solvent B in solvent A (solvent A: 0.1% formic acid, 2% ace-tonitrile; solvent B: 500 mM ammonium formate buffer pH 3.0, 2% acetonitrile) for 10 min, followed by 5–10%, 10–15%, 15–20%, 20–30%, 30–50%, 50–100%, and 100% solvent B in solvent A, each for 10 min at a flow rate of 4000 nL/min. Eluent from each gradient segment was pooled and injected into the trap column and then the analytical column using a gradient of 0–50% solvent D in solvent C over 60 min (solvent C: 0.1% TFA, 2% acetonitrile; solvent D: 0.1% TFA, 70% acetonitrile) and 50-100% solvent D in solvent C for 10 min at a flow rate of 300 nL/min. The RP column effluent was mixed with the MALDI matrix solution (4 mg/mL α-cyano-4-hydroxycinnamic acid dissolved in 70% acetonitrile containing 0.1% TFA and 80 μg/mL dibasic ammonium citrate) flowing at a rate of 1400 nL/min at the outlet, and spotted directly onto the ABI 4800 MALDI-plates in a 24 × 16 array at a frequency of two spots per minute. Four MALDI-plates corresponding to eight gradient segments in SCX chromatography (2736 spots) were analyzed.

### 2.3 MS and database search

Mass spectra were acquired in batch mode using an ABI4800 Proteomics Analyzer operated by 4000 Series Explorer Software (V. 3.5; ABI). Measurement conditions were set according to the values recommended by the manufacturer with some modification; the maximum number of peaks for MS/MS acquisition per spot, the S/N filter, and spot-to-spot tolerance were set at 10,100 or 150, and 200 ppm, respectively. For each MALDI plate, the instrument was set to scan mass spectra between 800 and 4000 *m/z*. Two or three cycles of non-redundant MS/MS scans were performed.

All MS/MS spectra were combined and a single database search was performed against primate protein sequences in the Swiss-Prot database using GPS Explorer software (V. 3.6; ABI) and MASCOT as a search algorithm. Search parameters included modifications by iTRAQT™ at N-terminal residues and internal K and Y residues, me-thylmethane thiosulphation of cysteine, and one missed cleavage; the maximum peptide rank was set at 2, and the minimum ion score was set at 85%.

### 2.4 Data analysis

Relative quantification of identical peptides labeled with four different iTRAQT™ tags was performed on the MS/MS peak list by comparing the areas of four signature peaks at 114,115,116, and 117 *m/z*. Values were normalized using the sum of values obtained for all tagged peptide pairs in the sample; data sets were imported into MS-Excel for further analysis. Relative changes in the abundance of individual peptides were defined as follows [S_11x_ (x = 4, 5, 6, or 7) and R represent normalized iTRAQT™ signal values and the ratio, respectively]: if R ≥ 1 (0 < R < 1) for any combination of S_115_ or S_117_/S_114_ or S_116_, the minimum (the inverse of the maximum) was adopted, representing a fold increase (decrease); otherwise, relative change was expressed as 1.0, meaning that detected changes were not consistent within internally replicated comparisons. Peptides with relative change values greater than 1.10 were subjected to manual inspection of the quality of their MS/MS spectra. MS/MS spectra without a complete set of four measurable iTRAQT™ tags were excluded from the analysis.

### 2.5 Western blotting and antibodies

Cell lysates prepared as described in Section 2.1 were separated by SDS-PAGE and transferred to PVDF membranes (Millipore, Japan) as previously described. The membranes were probed with a series of primary antibodies in combination with appropriate secondary antibodies conjugated to horseradish peroxidase (POD), and were visualized using POD immunostaining kit (Wako, Japan). The primary antibodies were: p94-specific anti-IS2 antibody, which was raised in goats [26–28], goat anti-eEF2 antibody (sc-13004, Santa-Cruz Biotech), mouse anti-β-actin antibody (A1978, Sigma, MO, USA), mouse anti-vimentin antibody (V6630, Sigma), goat anti-filamin A antibody (F2762, Sigma), mouse anti-fodrin mAb (clone AA6, Biohit, Finland), and mouse anti-annexin A1 and A2 antibodies (clones 29 and 5, respectively, BD Biosciences, USA). A polyclonal anti-GMMPR antiserum, which specifically detects the 150K fodrin fragment proteolyzed by calpain, was elicited using rabbits as previously described [33].

### 2.6 *In vitro* translation

*In vitro* translation experiments were performed as previously described using a rabbit reticulocyte lysate *in vitro* translation kit (DuPont-NEN, UK) and L-[^35^S]Met in the presence or absence of 380 μg/mL leupeptin and E-64 as previously described [26]. Briefly, the translation reaction mixture was mixed directly with an equal volume of SDS-PAGE sample buffer at specified times, and subjected to SDS-PAGE. Gels were dried, exposed to Fujix (Japan) imaging plates and X-ray films, and measured and analyzed using a Fujix Model BAS2000 Bioimage Analyzer.

## 3 Results and discussion

In COS7 cells, overexpressed p94 disappears very rapidly because of its autolytic activity (see below) [26] and simultaneously causes specific proteolysis of other proteins such as calpastatin and fodrin [17]. Calpastatin is a specific endogenous inhibitor of conventional calpains and fodrin is an *in vivo* substrate of conventional calpains. To identify proteins targeted by p94 protease activity, quantitative proteomic analysis of COS7 cells expressing either p94 WT or CS was performed using the iTRAQ™ technique and 2-D LC-MALDI-MS/MS analysis. Equal amounts of trypsin-digested peptides from each sample were individually labeled using one of four iTRAQT™ tags, which are specifically incorporated into free amine groups. As these tags are isobaric, have the same mass, and are chemically similar to one another, identical peptides originating from different types of cells were expected to be present in the same fractions throughout the LC separation procedure [29, 30]. Fragmentation of these peptides by MS/MS analysis simultaneously yields four specific peaks derived from each iTRAQT™ tag at 114, 115, 116, and 117 *m/z*; the intensity of the signal corresponds to the amount of peptides labeled with each tag. Thus, the relative abundance of identical peptides in different populations of cells, either WT or CS, was determined. Consequently, the amount of the parent protein from which the peptides were derived can be quantified. Integration of this information facilitates proteomic evaluation of the effect of p94 protease activity on intracellular proteins of culture cells in which conditions close to, if not equal to, that of the *in vivo* situation prevail. Proteins whose cellular abundance has been altered by p94 protease activity are expected to yield R values greater (down-regulated) or smaller (up-regulated) than 1 (no change), where R represents the ratio of iTRAQT™ signals for CS/WT. The comparison between WT and CS samples was performed using four R values (Fig. 1A).

**Figure 2:**
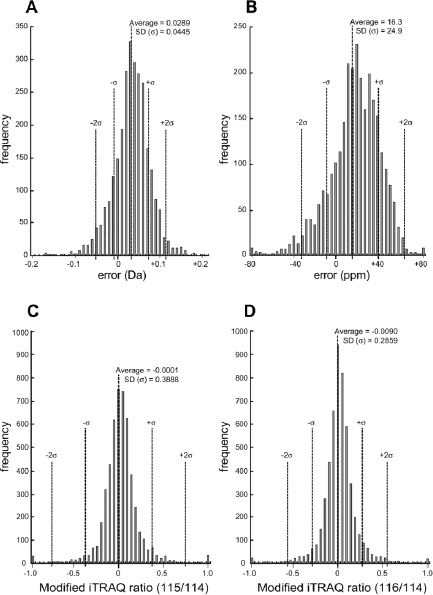
Statistical profiles of LC-MALDI analysis. (A, B) Error distribution of masses in Da (A) or ppm (B) between observed and calculated/theoretical molecular masses of 2801 identified MS/MS spectra out of 51 97 spectra. Dotted lines indicate means and ranges for one and two SDs. (C, D) Modified ratios of iTRAQT™ signals of 115 (CS-1) to 114 (WT-1) (C) or 116 (WT-2) to 114 (WT-1) (D). Values of ratios were modified as follows: Modified ratio = R − 1 (if R ≥ 1), 1/R − 1 (if 1 > R > 0), where R = iTRAQT™ signal ratio.

Using a total of 144 μg protein extracted from COS7 cells (36 μg labeled with each iTRAQT™ reagent), 2926 peptides, of which 1610 were unique, were identified from 5197 MS/MS spectra. Based on MS spectra of elution profiles of 2-D LC, visual inspection of several MS/MS spectra, and statistical assessment of iTRAQT™ signals (Figs. 2A and B), it was concluded that the experimental procedure yielded a reasonable amount of MS/MS data with reliability, and further analysis of the proteome of COS7 cells expressing p94 was performed. The identified peptides corresponded to 593 proteins, more than half of which were represented by a single unique peptide (Fig. 1B). As expected, abundant proteins were associated with many different peptides: vimentin, 23 peptides; HSP90β, 20 peptides; and β-actin, 14 peptides. Five peptides, 4 of which were unique, were identified for p94, which illustrates the difference between the sensitivity of this approach and that of other conventional methods such as Western blot analysis (see subsequent results).

**Figure 3:**
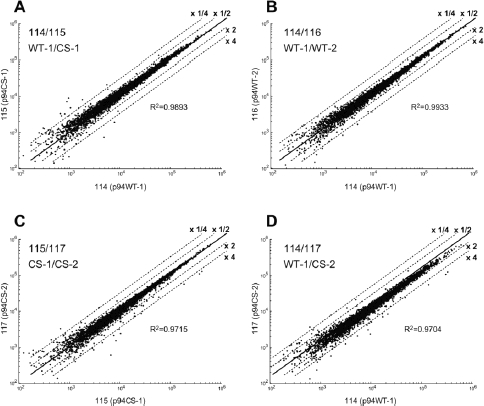
Scatter plots of iTRAQT™ signals (absolute values) between 114 (WT-1) and 115 (CS-1) (A), 114 (WT-1) and 116 (WT-2) (B), 115 (CS-1) and 117 (CS-2) (C), and 114 (WT-1) and 117 (CS-2) (D). Ranges of twofold and fourfold, one-half, and one-quarter are indicated by dotted lines. *R*^2^, correlation coefficient.

Distributions of modified signal ratios of iTRAQT™ 116 (WT-2) to 114 (WT-1) and those of 115 (CS-1) to 114 (WT-1) were compared to each other. Replicate WT samples showed that signal measurement was reproducible (mean = −0.0090, SD = 0.2359) (Fig. 2D). Although most peptides were expressed at similar levels in WT and CS samples, the SD was larger for this pair, indicating that peptides whose abundance differed between WT and CS were present (Fig. 2C). These properties are also evident in the scatter plots of iTRAQT™ signals for each of WT-WT and WT-CS pairs (Figs. 3A and B). The reproducibility of data for WT samples was in good accordance with the expected accuracy of this method [34], while replicated analysis of CS samples resulted in slightly poorer repro-ducibility than that of WT (Figs. 3C and D). To minimize false positives, only peptides for which the iTRAQT™ signal ratios (Rs) of all possible combinations of WT and CS samples, *i.e.*, 115/114, 117/114, 115/116, and 117/116, changed in identical directions were considered for further analysis.

**Figure 4:**
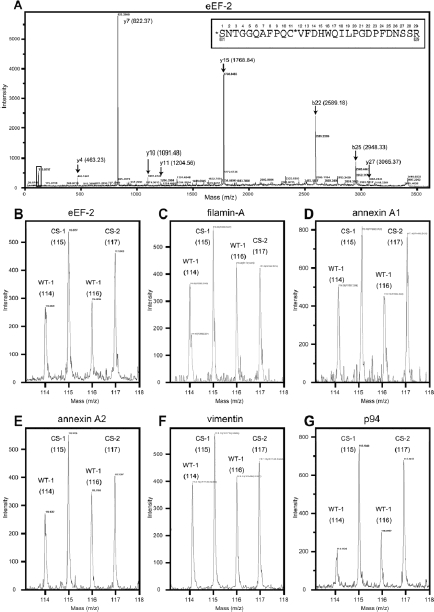
Examples of MS/MS spectra that exhibited significant differences in iTRAQT™ signal ratios. (A) MS/MS spectrum of precursor ion of 3410.5859, which was identified as elongation factor 2 with ion score 72 and I.C. 99.9% [calculated mass is 3410.5439, error = 0.0420 (1 2 ppm)]. Representative peaks are shown and calculated masses are indicated in parentheses. The predicted amino acid sequence is shown in the box, which corresponds to residue 801–829 of human EF2 with N-terminal iTRAQT™ and Cys-MMTS modification (indicated by *). The small square around *m/z*= 100 is the area magnified in (B). (B) Magnification of iTRAQ™ signals shown in (A). (C–G) Magnified iTRAQT™ signals of MS/MS spectrum (data not shown) identified as filamin A (C), annexin A1 (D), annexin A2 (E), vimentin (F), and p94 (G). The predicted peptide corresponds to residues 309–330 of human filamin A, 29–52 of human annexin A1, 1 79–195 of human annexin A2, 36–49 of human vimentin, and 733—748 of human p94.

Extracted peptides were sorted according to the relative change in abundance (= R_min_ if R ≥ 1; = 1/R_max_, if 1 > R > 0, where R = signal ratio of 115 or 117 to 114 or 116) (Table 1). In several studies conducted using the iTRAQ™ methodology, differences greater than 1.2-fold have been suggested to be considered significant [35]. In the present analysis, where a difference is represented by the minimal fold change, 1.10 was used as an arbitrary cut-off point. MS/MS spectra of these peptides were inspected visually. Proteins represented by peptides with reliable MS/MS spectra and whose signal ratios were decreased by WT p94 expression are listed in Table 2. Representative images of MS/MS spectra for amino acid sequencing and iTRAQT™ signal quantification are shown in Fig. 4.

**Table 1:** Relative abundance quantified by iTRAQT™ analysis

	Increased by p94 (WT > CS)			Decreased by p94 (WT < CS)			Total
Relative change	>1.25	>1.15	>1.10	1.10<	1.15<	1.25<	
No. of peptides	22	48	76	80	52	29	307
No. of proteinsa)	20	40	61	59	44	22	246
No. of proteinsb)	5	2	15	10	10	3	45

a) Number of proteins represented by identified peptides.

b) Number was obtained using averaged iTRAQT™ ratios for all peptides representing a protein.

**Figure 5:**
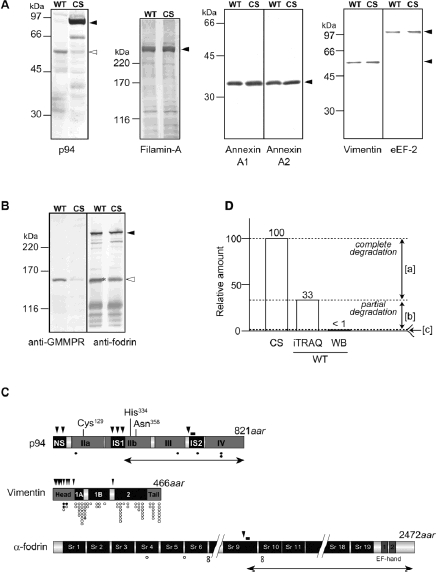
Comparison of identified proteins by iTRAQ™ analysis. (A) Western blot analysis of selected proteins using specific antibodies. Equal amounts of protein extract from COS7 cells expressing either WT or CS mutant of p94 were loaded for each blot. Closed and open arrowheads indicate the full-length and the autolyzed fragments, respectively, detected by anti-pIS2 antibody. The epitope region is indicated by a vertical bar in (C). AnxA1, annexin A1; AnxA2, annexin A2. (B) 150K fragment of α-fodrin proteolyzed at the calpain-specific site detected by anti-GMMPR in a protein sample from COS7 cells expressing WT but not CS (open arrowhead). The anti-fodrin mAb detected several fragments of different sizes in addition to full-length unproteolyzed fodrin (closed arrowhead) in both the WT and CS lanes. *, Proteolyzed 150K fragment specific to the WT sample was also detected by the anti-fodrin mAb, but the signal overlaps with an unidentified band with an almost identical mobility, which was also present in the CS sample. (C) Positional relationships between previously determined proteolytic sites and peptides identified in the present analysis. The closed arrowheads indicate autolytic sites for p94 and proteolytic sites of conventional calpains for vimentin and α-fodrin. Closed, open, and gray circles indicate the positions in proteins of peptides whose relative abundance were decreased, unchanged, or increased, respectively, in COS7 cells expressing WT p94 relative to cells expressing p94:CS. Cys, His, and Asn indicate catalytic amino acid residues in p94. Vertical bars indicate the positions of epitopes for anti-pIS2 (for p94) and anti-GMMPR (for the calpain-specific 150K proteolytic fragment of fodrin). Bidirectional arrows indicate the autolytic fragment of p94 and the calpain specific proteolytic fragment of fodrin, which correspond to the open arrowheads in (A) and (B), respectively. NS, IS1, and IS2 are p94-specific insertion sequences. IIa and IIb comprise the calpain protease domain. III and IV are C2-like and 5EF-hand motif Ca^2+^-binding domains, respectively. 1A, 1 B, and 2, coiled coil domains. Sr1-19, spectrin repeat. (D) Relative quantities of p94 protein/peptide by iTRAQT™ method and Western blot analysis (WB). WT p94 amounts detected by both methods are represented as relative to those of CS (CS = 100). Difference between CS and WT by iTRAQ ([a]) corresponds to portions in WT degraded completely (to amino acid) or at least smaller than the length of trypsin peptides. Difference between iTRAQ and WB in WT ([b]) corresponds to portions in WT degraded partially, larger than the length oftrypsin peptides but small enough not to be detected by WB. Region [c] mainly corresponds to the 55-kDa band in WB (A, “p94”, open arrowhead).

Among the proteins listed in Table 2, p94 was ranked as the protein with the greatest decrease (averaged R_min_ for peptides = 3.024, *n* = 5) (Fig. 4G). This is the first quantitative demonstration that the best substrate for p94 is p94 itself. The data also illustrate that the meaning of a quantitative change of a protein determined by the abundance of peptide derivatives is different from that detected by other methods such as Western blot analysis. An au-tolytic decrease in the full-length p94 in COS7 cells was detected by Western blot analysis, which showed that the amount of WT (addition of 94 kDa, 55 kDa and other smaller bands) is less than 1/100 of that of CS (Fig. 5A “p94”). On the other hand, the quantity of WT p94 was *ca*. 1/3 of that of CS at the peptide level when measured by the iTRAQT™ method (Table 2, Fig. 5D). All five (four unique) peptides (Fig. 5C "p94") detected for p94 decreased in WT samples to a similar extent. This large difference of detected relative WT quantity suggests that most of identified peptides for WT p94 by the iTRAQT™ method are derived from extensively autolyzed fragments of p94 that could not be detected by Western blot analysis due to small molecular mass and heterogeneity of the autolyzed fragments (Fig. 5D). Similar decreases of all five peptides detected for WT p94 indicate that upon autolysis, different autolyzed fragments of WT p94 concurrently become more susceptible to subsequent degradation than unau-tolyzed p94 protein.

**Figure 6:**
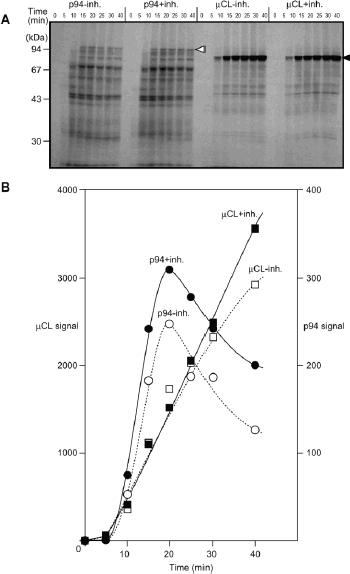
Time course of *in vitro* translation of p94 and the μ-calpain large subunit (μCL). *In vitro* transcribed RNAfor rat p94 and human μCL were translated with rabbit reticulocyte lysate and [^35^S]Met in the presence (+inh.) or absence (−inh.) of 380 μg/mL leupeptin and E64, and sampled at the indicated time (min). Samples are separated by SDS-PAGE, and exposed to X-ray film (A) and an imaging plate. The radioactivities of 94-kDa (open triangle) and 80-kDa (closed triangle) bands were quantified using a Fujix Bioimage Analyzer, and plotted against the time of sampling (B). Note that the scales for the Y-axes differ by a factor of 10.

Some of the proteins listed in Table 2 are previously identified substrates for other calpains [36–39] (see the “Previous report” column in Table 2). At present, it is not biochemically confirmed that the identified proteins are direct substrates for p94. Together with the previous observation, however, that p94 and the conventional calpains have some common substrate specificities [28], the present results are indicative for molecular context where p94 directly exerts its activity. The identification of filamin-A and vimentin as substrates is consistent with the observation that ectopic expression of p94 in several different culture cells causes morphological defects accompanied by proteolysis of several cytoskeletal proteins, including filamin-A and fodrin [40]. Western blot analysis detected a slight decrease in filamin-A and vimentin in WT samples, while the decrease in annexin A1 or A2 was less obvious (Fig. 5A). The proteins listed in Table 2 also include components of translation machinery (eEF1α-1 and -2, eEF-2 and initiation factor 3). eEF-1α is proteolyzed by drosophila calpain [36], and eEF-2, which was shown to be related to p94, was decreased in WT samples according to Western blot analysis (Fig. 5A). These results strongly suggest that there is interaction between p94 and the translation machinery, which was further confirmed by the following experiment. When p94 was translated *in vitro* using the rabbit reticulocyte lysate system, p94 exhibited co-translational autolytic activity during the initial phase; the signal intensity for the full-length translated product was *ca*. 1/10 lower than that of the μ-calpain large subunit (μCL) translated in the same system (Fig. 6A, 0–20 min). The intensity of the 94-kDa band as well as that of other smaller bands reached a local maximum at about 20 min from the start of translation and then decayed (Fig. 6B). In contrast, μCL exhibited an almost linear increase in 80-kDa band intensity regardless of the presence of high concentrations of the Ser/Cys-protease inhibitors, leupeptin and E64 (Fig. 6, + or −inh.). If p94 only had autolytic activity, the amount of p94 would not have decayed, but would have asymptotically approached a plateau at which protein synthesis would have been in balance with autolytic degradation. Thus, the observed local maximum with subsequent decay indicates that synthesis of p94 protein gradually decreased, which was probably due to proteolysis of the translation machinery by p94 as shown in iTRAQT™ experiments. These data strongly suggest that p94 is involved in regulation of the eukaryote protein synthesis system, as is the case for the conventional calpains [36].

**Table 2:** Selected proteins with CS/WT abundance ratios greater than 1a)

Rank	Protein	Entry	Swiss-Prot acc. no.	*M*_r_	No. of iTRAQ™ peptides with R_min_ >1.10	Mean of R_min_	No. of unique peptides	No. of iTRAQ™ peptides	Total ion Score		Best Ion Score		Previous report	
										C.I.%		C.I.%	p94	μ, m-calpain
1	P94 / calpain3	CAN3_HUMAN	P20807	94 254	5	3.02	4	5	193.98	100.00	89.06	100.00	[26]	
2	Eukaryotic elongation factor 1-alpha 2	EF1A2_HUMAN	Q05639	50 470	2	1.65	7	13	320.85	100.00	64.72	99.99		[36]
3	Eukaryotic translation initiation factor 3 subunit 7	IF37_HUMAN	O15371	63 973	1	1.48	1	1	60.90	99.98	63.14	99.99		
4	26S Proteasome regulatory subunit 6A	PRS6A_HUMAN	P17980	49 204	1	1.34	1	1	71.36	100.00	72.89	100.00		
5	Filamin-A	FLNA_HUMAN	P21333	280 608	3	1.31	10	21	824.85	100.00	144.35	100.00	[40]	[38]b)
6	Importin-7	IPO7_HUMAN	O95373	119 517	1	1.28	2	5	197.07	100.00	100.31	100.00		
7	Eukaryotic elongation factor 2	EF2_HUMAN	P13639	95 207	3	1.28	11	16	749.70	100.00	131.15	100.00		
8	Vimentin	VIME_HUMAN	P08670	53 520	5	1.25	23	74	1694.43	100.00	156.66	100.00		[37]b)
9	Annexin A1	ANXA1_HUMAN	P04083	38 583	2	1.23	2	6	218.54	100.00	188.19	100.00		[39]b)
10	Eukaryotic elongation factor 1-alpha 1	EF1A1_HUMAN	P68104	50 141	3	1.22	12	35	910.71	100.00	194.53	100.00		[36]
11	Annexin A7	ANXA7_HUMAN	P20073	52 739	2	1.21	1	5	158.97	100.00	158.97	100.00		[39]c)
12	Annexin A2	ANXA2_HUMAN	P07355	38 473	2	1.19	16	55	1470.31	100.00	164.31	100.00		[39]c)
13	Fructose bisphos-phatealdolase A	ALDOA_HUMAN	P04075	39 289	6	1.18	9	37	726.61	100.00	123.91	100.00		[47]c)
14	Thioredoxin	THIO_HUMAN	P10599	11 606	4	1.16	2	16	127.21	100.00	66.75	100.00		
15	Glyceraldehyde-3-phosphate dehydrogenase	G3P_HUMAN	P04406	35 922	3	1.15	8	26	779.77	100.00	214.62	100.00		
16	Triosephosphate isomerase	TPIS_HUMAN	P60174	26 538	4	1.14	6	16	501.27	100.00	119.92	100.00		

a) Identified proteins were ranked based on their increase in CS samples; a high rank corresponds to a high ratio of CS/WT, which implies a decrease in WT expression. R_min_, the minimum value for the iTRAQT™ signal ratio of1 15 or 11 7 to 114 of 116.

b) Proteolytic sites were determined.

c) Indicative observations are reported.

Other proteins identified as possible p94 substrates revealed novel aspects of the function of p94. Fructose bisphosphate aldolase A, glyceraldehyde-3-phosphate (GAP) dehydrogenase, and triosephosphate isomerase catalyze successive reactions (fructose bisphosphate → dihydroxy acetonephosphate + GAP → 2GAP → 1,3-bisphosphoglyceric acid) in the glycolytic system, which is highly developed in skeletal muscle. Another proteomic study using 2-DE and transgenic mice overexpressing p94 identified other glycolytic enzymes as p94 substrates in skeletal muscle [41], suggesting a role for p94 in the glycolytic cycle [42]. Importin-7 and the annexins are involved in membrane trafficking. As yeast calpain utilizes a membrane-associated scaffold, a similar mechanism might apply to p94 [43, 44]. Proteolysis of proteasome regulatory subunit RPS6A by p94 may indicate that the ubiquitin proteasome system is subject to regulation by calpain, at least in certain cases [45]. At present, the relationship between p94 and thioredoxin is unknown. It has yet to be clarified that cleavages of proteins identified in this study are characterized under physiological context as well as biochemically. It is particularly important to distinguish direct p94 substrates from those proteolyzed by other proteases activated by overexpression of p94. The latter case could non-physiologically happen in COS7 cells, since regulatory mechanisms for p94 activity, which are probably effective in skeletal muscle tissues, may absent here. It should also be noticed that proteins identified from COS7 cells could be alternatives for their counterparts expressed in skeletal muscle tissues. Integrated understanding of these issues would reveal physiological behavior of p94 and its relevance to other cellular machineries with more clarity.

A notable difference between p94 and the other proteins listed in Table 2 is that proteins other than p94, if represented by multiple peptides, were only decreased by a portion of their peptides. This is probably because the stabilities of products generated from substrate proteins by p94 differ. Figure 5C illustrates the distributions of the identified peptides and the reported proteolytic sites of p94, vimentin, and fodrin. While autolysis causes down-regulation of p94 as a whole molecule, proteolysis of vimentin would confer other properties on certain parts of the molecule. This fits a scheme in which calpains do not degrade target proteins, but modify the functions of targets. Consistent with this hypothesis, proteolysis of α-fo-drin was detected in WT-expressing cells by an antibody against a calpain-specific proteolytic product (Fig. 5B, anti-GMMPR) [17], but not by iTRAQT™ analysis. Six independent peptides from α-fodrin before and after the cal-pain cleavage site were detected by MS/MS (Fig. 5C), essentially none of which showed a change in relative abundance between WT and CS samples. This indicates that fodrin proteolysis by p94 did not change the total amount of fodrin. This quantitative information, which is hard to obtain solely by Western blot analysis (Fig. 5B, anti-fodrin), suggests that limited proteolysis of fodrin by p94 does not cause immediate degradation of fodrin fragments. Although the possibility of interference at a tran-scriptional/translational level as a consequence of p94 protease activity cannot be excluded, a more extreme change in the abundance of target proteins as a whole would have been observed if that were the case.

Western blot analysis showed that the decrease in identified proteins was rather small compared with the autolytic decrease in p94 (Fig. 5A). This was probably caused in part by much less efficient protein proteolysis by p94 than p94 autolysis, which could be because of that COS7 cells but not skeletal muscle cells were used. Although the expected advantage of our COS7 system is that ectopically expressed p94WT is the only active cal-pain present when cells were not otherwise stimulated, the lack of stimuli to spike signaling cascades such as [Ca^2+^]_i_ regulation or cytoskeletal motility might hamper contact between p94 and substrates and/or other molecules and enhance autolytic rather than proteolytic reaction.

Levels of several proteins were increased in WT samples (Table 1). This might indicate that proteolysis by p94 removes factors restricting the stability of proteins. If these proteins are p94 substrates, structural changes induced by proteolysis may make them more stable. On the other hand, enzymes that promote degradation of these proteins may be substrates for p94. Peptides with a fold-change greater than 1.1 were subjected to manual inspection of their MS/MS spectra. Several peptides had reliable MS/MS spectra, but few represented parental proteins with consistency. Thus, it was difficult to extract proteins whose increase in abundance was dependent on p94 protease activity. It is also possible that the protease activity of p94 was not responsible for increasing the abundance of other proteins. Identification of such proteins from COS7 cells was not pursued further and left for the future investigation using different cell culture system.

## 4 Concluding remarks

Quantitative comparison of proteomic data obtained from COS7 cells expressing p94WT or p94CS was efficient in surveying the change of protein abundance caused by p94 protease activity. The advantages of this method over the 2-DE method are not only simplicity and reproducibil-ity, but also the ability to manipulate high molecular weight proteins as evidenced by the results presented in Table 2 for filamin-A and importin-7. Simultaneous identification and quantification by iTRAQT™ analysis enabled us to detect potential p94 substrates that might have been overlooked because of a lack of information and/or analytical tools. Another advantage of the methodology described in this study is that it enables evaluation of the consequences of proteolytic events from the abundance of various proteolytic fragments from the same protein. As calpains have been implicated in modulation of the function of substrate proteins, the abundance of the parts of substrate proteins responsible for their functions does not necessarily decrease upon proteolysis. The quantitative nature and the unbiased sensitivity of the method will be useful for identifying the fates of various proteolytic products, which will complement other biochemical and cell biological studies of proteolysis [46]. This methodology can be applied to all calpain species in various differential analyses of pathological states, trans-genic mice, and developmental stages.

## Abbreviations:

CS: protease-inactive p94:C129S mutant
EF: elongation factor
iTRAQ: isobaric tags for relative and absolute quantitation
p94: skeletal-muscle-specific calpain, calpain 3
LGMD2A: limb-girdle muscular dystrophy type 2A
WT: wild type
